# Letter from the Editor in Chief

**DOI:** 10.19102/icrm.2026.17094

**Published:** 2026-09-15

**Authors:** Devi Nair


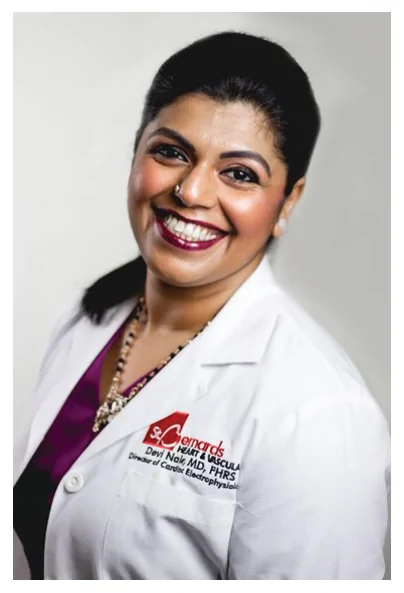


Dear Colleagues,

Welcome to the September 2026 issue of *The Journal of Innovations in Cardiac Rhythm Management*.

In August, I wrote that I was looking forward to HRX Live, and this month I write having lived it. Our community gathered in Atlanta from September 18 through 20 for a meeting built, uniquely among our gatherings, around the disruptive edge of the field rather than the incremental refinement of established technique. HRX opened with the Innovation Summit, which I had the privilege of co-producing: a fast-moving half-day devoted to the technologies just now arriving at the bedside, from smarter cardiac mapping and next-generation ablation to breakthrough devices and pacing and the rapidly maturing role of artificial intelligence in diagnosis and triage. What made the Summit worthwhile was not the parade of new tools but the room itself: clinicians, engineers, entrepreneurs, and regulators pressed into the same conversation, each forced to defend assumptions the others did not share. Innovation in our field has never come from any one of those constituencies alone, and the most valuable hours were the ones in which they disagreed productively about what a technology must prove before it earns a place in our laboratories.

The main stage reinforced the point. This year’s plenary sessions were built around three figures who together trace the arc of our field. Murali Aravamudan spoke to the promise of artificial intelligence and to how much of the diagnostic reasoning we currently do by hand may soon be augmented by machines. Rebecca Seidel, speaking from industry, was a reminder that the road from a good idea to a usable device runs through engineering, manufacturing, and regulation and that none of these steps is incidental. And Michel Haïssaguerre, whose recognition of pulmonary vein triggers remade the treatment of atrial fibrillation, stood as living proof that today’s disruptive idea becomes tomorrow’s standard of care, a through-line that runs directly into several of the manuscripts in this very issue.

Planning for next year is already underway. HRX Live 2027 will convene in Dallas from September 17 through 19, and I am honored to serve as a co-producer alongside Prashanthan Sanders, with the leadership of the Heart Rhythm Society joining us. To those of you who were with us in Atlanta this year, thank you; to those who were not, consider this an early and warm invitation. The conversation we began in Atlanta is only going to deepen, and it is at its best when the whole of our community is in the room.

## In This Issue

The manuscripts in this issue read almost as a companion to that conversation. They move from real-world outcomes with a novel energy source to the widening frontier of ventricular tachycardia ablation and left atrial appendage closure, a fluoroless technique that pushes mapping to its limits, and finally a cautionary report that reminds us innovation and vigilance must travel together.

In an original research contribution, Sabayon, Abu Jazar, and colleagues^1^ from the University of Texas Medical Branch report a prospective, single-center experience with pulsed field ablation using the Farapulse™ system (Boston Scientific, Marlborough, MA, USA) in 100 consecutive patients with atrial fibrillation. With median procedure and fluoroscopy times of 42 and 12 min, no major adverse events, and 82% freedom from recurrence off anti-arrhythmic drugs at 6 months, their real-world data add welcome, if short-term, support to the broader adoption of this technology.

Mohamed, Ghaleb, Kolawole, and Khan^2^ offer a comprehensive review of the global advances in ventricular tachycardia ablation, tracing the arc from early surgical and direct-current approaches to today’s multimodal landscape: refined radiofrequency, pulsed field, and cryoablation; remote magnetic navigation; autonomic neuromodulation; and the noninvasive promise of stereotactic body radiation therapy. It is a useful map of a field that increasingly demands more than one tool.

Zito, Horton Embrey, La Fazia, and colleagues^3^ trace the past, present, and future of left atrial appendage closure, from its surgical origins to contemporary transcatheter devices, the evidence against warfarin and the direct oral anticoagulants, and the design advances aimed at reducing peri-device leak and device-related thrombus. As indications widen, their review marks a therapy moving from niche intervention toward the mainstream of stroke prevention.

Costa, Hakim, and colleagues^4^ from Henry Ford Genesys describe what is, to their knowledge, the first fluoroless ablation of a coronary sinus–related epicardial accessory pathway performed without coronary sinus venography, in a woman with two prior failed procedures. Relying solely on three-dimensional electroanatomic mapping, they achieved durable success at 1 year, a striking illustration of how far mapping alone can now carry a complex case while sparing radiation and contrast.

Finally, Golzarian, Kanjwal, and Hakim^5^ report a transient ischemic attack caused by delamination of a steerable guiding sheath during atrial fibrillation ablation, a thromboembolic complication traced to a failure in the device’s structural integrity. It is a sobering counterpoint to the rest of the issue and a reminder that every new tool we bring into the laboratory carries failure modes we are still learning to anticipate.

## In Closing

If there is a thread running from the Innovation Summit through these pages, it is that progress in our field is never self-justifying; each advance must earn its place through evidence, scrutiny, and honest reporting of what goes wrong as well as what goes right. I will close, as ever, with an invitation. If you have a study, a review, a case, or a tracing that advances that conversation, this journal exists to publish it, and we are always glad to see the work of trainees and of colleagues from every corner of our global community.

I am grateful, as always, to our authors, reviewers, and editorial team for their work, and to you, our readers, for your continued engagement with the journal.

Warm regards,


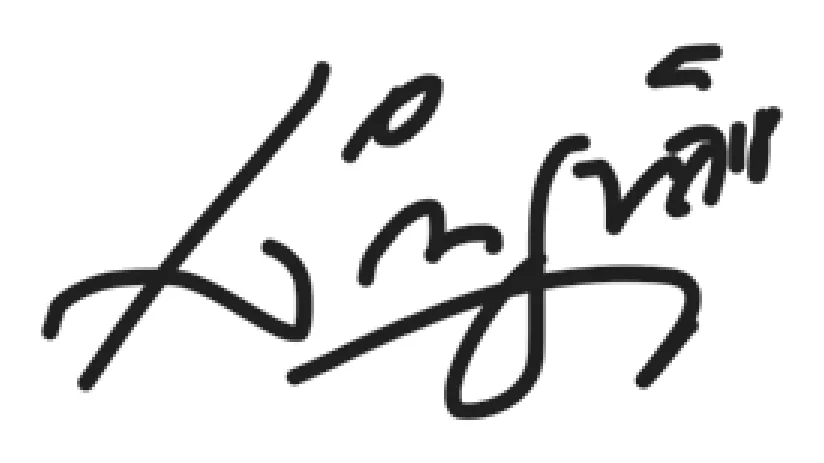


Dr. Devi Nair, md, facc, fhrs

Editor-in-Chief

*The Journal of Innovations in Cardiac Rhythm Management*

Director of the Cardiac Electrophysiology & Research,

St. Bernard’s Heart & Vascular Center, Jonesboro, AR, USA

White River Medical Center, Batesville, AR, USA

President/CEO, Arrhythmia Research Group

Clinical Adjunct Professor, University of Arkansas for Medical Sciences

Governor, Arkansas Chapter of the American College of Cardiology

drdgnair@gmail.com
